# Processing of Individual Items during Ensemble Coding of Facial Expressions

**DOI:** 10.3389/fpsyg.2016.01332

**Published:** 2016-09-07

**Authors:** Huiyun Li, Luyan Ji, Ke Tong, Naixin Ren, Wenfeng Chen, Chang Hong Liu, Xiaolan Fu

**Affiliations:** ^1^State Key Laboratory of Brain and Cognitive Science, Institute of Psychology, Chinese Academy of SciencesBeijing, China; ^2^University of Chinese Academy of SciencesBeijing, China; ^3^Department of Experimental Clinical and Health Psychology, Ghent UniversityGhent, Belgium; ^4^Department of Psychology, University of South Florida, TampaFL, USA; ^5^Department of Psychology, Bournemouth UniversityPoole, UK

**Keywords:** facial expression, emotion, individual representation, ensemble representation, processing resource, diffusion model

## Abstract

There is growing evidence that human observers are able to extract the mean emotion or other type of information from a set of faces. The most intriguing aspect of this phenomenon is that observers often fail to identify or form a representation for individual faces in a face set. However, most of these results were based on judgments under limited processing resource. We examined a wider range of exposure time and observed how the relationship between the extraction of a mean and representation of individual facial expressions would change. The results showed that with an exposure time of 50 ms for the faces, observers were more sensitive to mean representation over individual representation, replicating the typical findings in the literature. With longer exposure time, however, observers were able to extract both individual and mean representation more accurately. Furthermore, diffusion model analysis revealed that the mean representation is also more prone to suffer from the noise accumulated in redundant processing time and leads to a more conservative decision bias, whereas individual representations seem more resistant to this noise. Results suggest that the encoding of emotional information from multiple faces may take two forms: single face processing and crowd face processing.

## Introduction

While progress has been made in understanding facial expressions, most studies focused on the processing of an isolated emotional face. However, it is quite often that we encounter multiple faces with emotional expressions in real life scenarios. Do we process the facial expressions individually or as a whole, or both? The current study aims to investigate how individual faces are processed and their roles in ensemble coding of multiple facial expressions.

### Processing Multiple Facial Expressions

Due to the limited attentional resources and short-term memory capability (Luck and Vogel, 1997; Scholl and Pylyshyn, 1999), our visual system must compress the incoming visual input by efficient coding (Alvarez, 2011; Haberman and Whitney, 2012). Remarkably, the brain is good at encoding repeated and redundant visual information effortlessly by extracting a mean from similar visual features in a scene. This compresses redundant influx of information. It has been demonstrated that the brain is able to simplify and represent repeated similar patterns by an ensemble representation (e.g., mean) across different feature domains such as orientation, brightness (e.g., Watamaniuk et al., 1989; Dakin and Watt, 1997; Parkes et al., 2001; Bauer, 2009). Averaging such low-level features may have direct neural substrate. For example, when seeing a group of moving dots, neurons sensitive to specific directions in the visual cortex may be evoked in a parallel manner, thus integrate the moving direction of the multiple dots (Treue et al., 2000).

With the membership identification and mean discrimination paradigms, researchers has extended the mean representation research to other low-level features, e.g., size (Ariely, 2001), position (Morgan and Glennerster, 1991; Morgan et al., 2000; Alvarez and Oliva, 2008), speed (Watamaniuk et al., 1989; Watamaniuk and Duchon, 1992). In the membership identification paradigm, observers were first shown a set of items for a period of time and then were asked to identify whether a follow-up test item was a member of the previous set. If an observer achieved high accuracy in membership identification, then it can be inferred that the observer had obtained a precise individual representation of the items in the set. However, an intriguing result found by Ariely (2001) was that when the size of test circle approached the mean size of the previous set, observers would be more likely to report it as the member, even if it was actually not present previously. This suggested that observers implicitly formed a mean representation of the set and matched it with the test items. In the mean discrimination paradigm, observers were explicitly asked to compare the mean of the previous set of items with the test item. Ariely (2001) found that observers were able to discriminate the mean size of several circles with high accuracy, nearly as precise as discriminating the size of a single circle.

These findings attracted broad research interest toward representation of multiple items by means. Haberman and Whitney (2007) further extended the study to higher-level information (e.g., faces). They morphed images of two emotional expressions of a face to present set of facial expressions varying different levels of intensities between the two expressions. They then tested the observers’ ability to discriminate the mean emotion intensity from multiple morphs that contained different proportions of the two expressions. Results showed no significant difference between the thresholds for discriminating the emotion of a single face and the mean emotion of multiple faces, suggesting that human observers could rapidly extract the emotional information from a set of multiple images (Haberman and Whitney, 2007, 2009). This ability is not limited to simultaneously presented faces, observers are also able to extract emotion information from successively displayed faces (Haberman et al., 2009). In addition to facial expression, observers are also able to extract mean representations of gender (Haberman and Whitney, 2007), identity (de Fockert and Wolfenstein, 2009), race (Jung et al., 2013), biological motion (Sweeny et al., 2013), and gaze of crowd (Sweeny and Whitney, 2014).

### Poorer Individual Representations in Ensemble Coding

There is evidence that ensemble representation can be extracted very rapidly and efficiently, even when the visibility of individual items was diminished (Choo and Franconeri, 2010), or under conditions of reduced attention (Alvarez and Oliva, 2009; Joo et al., 2009). However, it remains unclear how these ensemble representations are computed (Alvarez, 2011), especially on the relationship between individual representations and ensemble coding. According to one hypothesis, an ensemble representation is computed without first build individual representations. We will call this the “element-independent assumption.” A main supporting evidence for this assumption is the possibility to compute accurate ensemble representations even the individual representations are impoverished or lost (Parkes et al., 2001; Alvarez and Oliva, 2009; Haberman and Whitney, 2009, 2012; Choo and Franconeri, 2010; Fischer and Whitney, 2011). For example, individual representation in a set of circles was rather imprecise while the mean size could be perceived precisely (Ariely, 2001). In accordance with the results from low-level membership identification studies, the recognition rate for individual member in a set of faces is low, indicating underdeveloped individual representations (Haberman and Whitney, 2007). In contrast, observers usually show a tendency to report faces with mean emotion intensity as a member (Haberman and Whitney, 2009). Consistent with this, ensemble representations of multiple objects are also better than representations of single individuals (Sweeny et al., 2013).

It remains unclear why recognition of individual items is poorer than ensemble representation. It has been suggested that perceptual similarity is the dominant factor determining the performance of ensemble coding (e.g., Utochkin and Tiurina, 2014; Maule and Franklin, 2015). In most studies of ensemble coding, the elements in the set are often so similar (e.g., morphed stimuli) that more resources and time are required to discriminate the stimuli from one another. However, sufficient resources might be unavailable in prior studies. This raises a possibility that the individual representations could be improved if resource limitation were minimized. There are at least two possible means to minimize resource limitation. One is to increase total available resource, the other is that stimuli require less processing resource. Indeed, Neumann et al. (2013) used celebrity faces to study mean identity representation and found that observers could form mean identities and also preserve precise representations of individual identities. The salience of celebrity identities, requiring less resource to process, may be the key to the stronger individual representations. This result provides evidence that the poor representation of individual items could be improved if required processing resources are sufficient. Moreover, if individual items are allocated with more resource, the individual representations would affect the ensemble coding to a more extent. For example, with more attention oriented to certain individuals, their weights in the mean representation would increase (de Fockert and Marchant, 2008). Wolfe et al. (2013) used an eye tracking technique to investigate attention allocation in multiple faces. Results showed that the faces with more eye gaze, indicating more resource allocated, occupied a higher weight in the mean representation. These results seem to imply that ensemble coding is not independent of the processing of individual representation when processing resource for individual items is sufficient.

Alvarez (2011) points out that a poor representation of individual items is not necessarily a consequence of mean computation without computing individuals. For example, Ariely (2001) suggests that the individual representations could be computed and then discarded. This has been supported by Fischer and Whitney (2011) who showed that although participants were unaware of the emotional expression of the central face in the set, it did impact the perceived mean emotion of the entire set. Another alternative possibility is that the individual representations are not discarded, but are simply so noisy and inaccurate such that observers cannot consistently identify individuals from the set owing to this high level of noise (see Alvarez, 2011, for a review). It has been suggested that the internal noise that limits the processing of ensemble representation is lower than that for a single object (Im and Halberda, 2013), because of the averaging process in which the noise of multiple individual measurements cancels each other out. The visual system can compensate for noisy local/individual representations by collapsing across those local features to represent the ensemble statistics. Taken together, it raises a possible contribution for ensemble coding from redundancy gain of individual items, which was found in the emotion processing of multiple faces in a brief presentation (200 ms,Won and Jiang, 2013). This element-dependent assumption suggests that the ensemble representation will benefit from the improvement of individual representations.

### Goals of the Current Study

The empirical evidence reviewed above indicates an important role of the processing resources for individual representations. This offers an alternative perspective on the relationship between individual representations and ensemble coding. Taken from this perspective, the current study aimed to investigate the roles of individual representation in processing multiple faces with varying processing resources. Haberman and Whitney (2009) has manipulated the set duration in a membership identification task, and found the representation of mean emotion becomes noisier as set duration decreases. However, they only focused on mean representation extracted implicitly, and didn’t report the data related to the different representation of set members and non-members. Furthermore, no study has addressed the processing resource issue in the mean emotion discrimination task where mean representation is required to extract explicitly. Thus, it remains unclear for the impact of set duration on the relationship of individual and mean emotion representations.

To demonstrate the role of resources in the processing of individual items, we manipulated the available processing time during the membership identification task (Experiment 1) and the mean discrimination task (Experiment 2) to examine its impact on the relationship of individual and mean representations. Our hypothesis was that the processing constraint of individual representations improves with more processing time available. Specifically, mean representation would be better than individual representation when little time available for processing multiple faces, but individual representation would be improved with sufficient time.

## Experiment 1

This experiment examined whether individual representation of a face set could be improved via greater processing time. We adopted the membership identification paradigm in which participants were asked to indicate whether the test face was a member of the previously presented set of faces. The length of presentation time was manipulated. The task was to judge whether the test face was a member of the face set or not. Participants were told to answer “yes” for members, “no” for non-members. We used two categories of faces for non-members, one was a face with the mean emotion intensity of the set, and the other was a face not shown in the set. Our hypothesis was that observers would be more accurate in the membership identification tasks as a function of presentation time. With longer presentation durations, participants should be able to recognize more actual members and rejecting more mean representations due to enhanced precision of individual representations. That is, the precision of individual processing would allow participants to discriminate individual representation better relative to the computation of mean representation.

### Methods

#### Participants

Twenty (age 18–25, 10 males) undergraduate and graduate students participated in this experiment for a small payment. All are right-handed and have normal or corrected-to-normal vision. The study was approved by the Institutional Ethics Review Board of the Institute of Psychology, Chinese Academy of Sciences. All participants were treated in accordance with the APA’s guidelines. Informed consent was obtained before the experiment.

#### Stimuli

**FIGURE 1:**
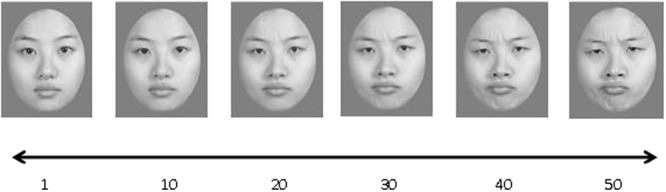
**Sample stimuli used in Experiments 1 and 2: A series of face images with gradual change in emotional intensity from neutral to disgust**.

Fifty face images were generated by morphing between two emotionally extreme faces of the same person (**Figure 1**), one of our young female lab members. The emotional expression among the faces ranged from neutral to disgust, with Face 50 being the most disgusted. The difference in emotion intensity between two contiguous images were denoted as one emotional unit, i.e., about 2% morph. All face images were rendered into grayscale and displayed on gray background. Each image extended a viewing angle of 3.78° × 4.82°. In each trial, four images were presented in a 2 × 2 invisible matrix, extending 8.78° × 10.16° in total.

Following Haberman and Whitney (2009), each stimuli set consisted of four images with different emotional intensity, differing at least six emotional units from each other, a distance above the participants’ discrimination thresholds. The mean values of the emotional intensity were randomly chosen from a pool of stimulus sets before each trial and the four images were given the values of mean ± 3 and mean ± 9. The mean changed on every trial but was never an element of the set. Test faces had three types: “member” were actual images in a stimulus set; “mean” was the mean emotional intensity of a stimulus set; “neither member nor mean” were images that had an emotional intensity of the mean ± 15, ±12, or ±6.

#### Procedure

**FIGURE 2:**
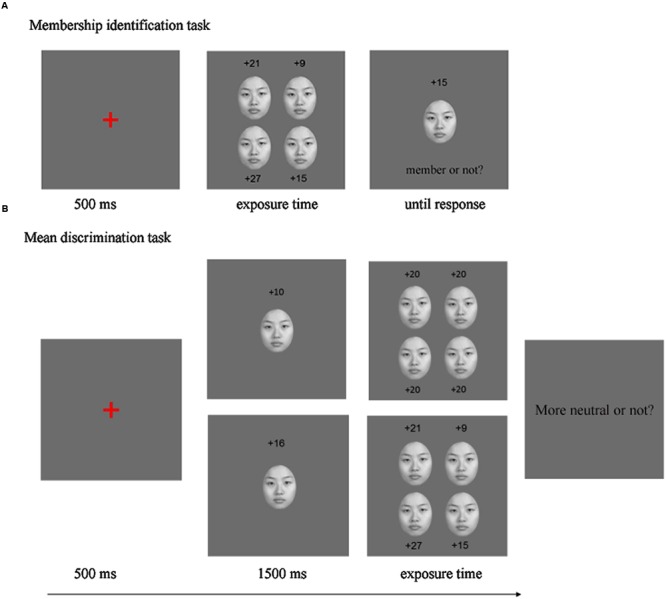
**Schematic procedure of Experiments 1 **(A)** and 2 **(B)****. Exposure time could be 50 /500/1000/1500/2000 ms (1500 ms excluded in Experiment 2). Top row of **(B)** depictured mean discrimination task for homogenous set, and bottom row of **(B)** for heterogeneous set. The numbers were given for illustration of emotional intensities, and not displayed in experimental trial.

Participants were seated 60 cm before the monitor. Instructions were given on the screen. In each trial, after a 500 ms fixation, the stimulus set was presented for a designated exposure time, followed by the test face (**Figure 2A**). Participants were asked to judge whether the test face was a member of the stimuli set (2AFC) and press the corresponding key. As defined in the stimuli section above, a test face was a “member” if it had previously been presented in the set. It was called a “non-member” otherwise. The test face was presented until the participant made a response.

Exposure time was manipulated in blocks, and three types of test faces were randomized in each block. The complete experiment had five blocks, each with 110 trials (40 Member, 10 Mean, and 60 Neither). The order of the blocks was counterbalanced between participants.

### Results

**FIGURE 3:**
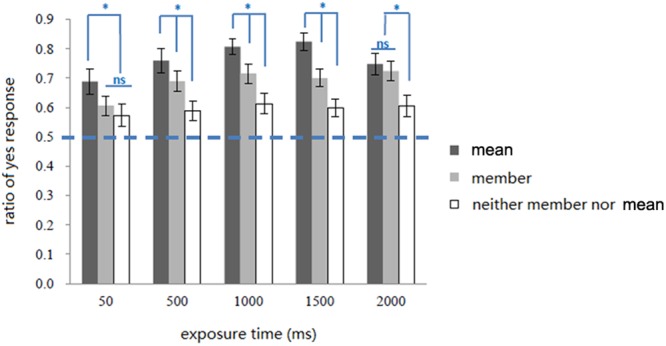
**Ratio of “yes” response in all conditions in Experiment 1.** Error bars represents one standard error of means, ^∗^ *p* < 0.05.

The trials where participants responded too quickly or slowly (more than two standard deviations below or above the mean RT) were excluded from further analysis. This resulted in an exclusion 1% of all trials. The data of ratio of “yes” response (**Figure 3**) were analyzed. A “yes” response indicates that participants thought that the test face was a member of the preceding set. The Cox-Small test was used to assess the multivariate normality, which showed that the data of “yes” response ratio were normally distributed (*p* = 0.430). A 3 (type of test face) × 5 (exposure time) repeated-measures ANOVA revealed significant main effects for type of test face *F*(2,38) = 51.31, *p* < 0.001, η_p_^2^ = 0.73; and exposure time *F*(4,76) = 4.02, *p* < 0.01, η_p_^2^ = 0.17. The interaction of the two factors was also significant *F*(8,152) = 2.76, *p* < 0.01, η_p_^2^ = 0.13.

Simple effect analysis showed that exposure time affected the ratio of “yes” responses to both types of test face Mean, *F*(4,76) = 3.26, *p* < 0.05, η_p_^2^ = 0.15, and Member, *F*(4,76) = 6.20, *p* < 0.001, η_p_^2^ = 0.25, but had no effect on responses to the Neither type, *F*(4,76) < 1, *p* > 0.05, η_p_^2^ = 0.05. Trend analysis showed varying responses to the test face types Mean and Member. The trend of responses to Mean as a function of exposure time was not linear, *F*(1,19) = 2.74, *p* > 0.05, where the “yes” responses ratio first increased and then decrease with longer exposure times. In contrast, the response trend for Member test faces as a function of exposure time showed were a linearly rising pattern, *F*(1,19) = 13.99, *p* < 0.01, η_p_^2^ = 0.42.

In addition, when a set of stimuli was shown for 50 ms, the ratio of “yes” response to Mean test faces was significantly higher than to Member and Neither test faces (*p*s < 0.05), while the latter two were not significantly different (*p* = 0.16). When a set of stimuli was shown for 500/1000/1500 ms, the ratio of “yes” response was higher for the Mean than for Member and both were higher than for Neither test faces (*p*s < 0.05). When a set of faces was shown for 2000 ms, there was no significant difference between responses for mean and Member (*p* = 0.31), but both were higher than Neither test faces (*p*s < 0.05).

### Discussion

When the set of faces were presented for only 50 ms, the ratios of “yes” response for Member and Neither conditions were not significantly different. Considering the stable difference between these two conditions with longer presentation durations, this result suggested that within a very brief visual exposure, participants were unable to process the details of the individual faces. However, with 50 ms exposure, participants made more “yes” responses to a set mean, which is consistent with that existing evidence for fast extraction of mean emotion information from multiple faces (Haberman and Whitney, 2009). The result supports the idea that mean representation could be formed without precise individual representation.

When exposure time was up to 500 ms, the ratio of “yes” response to “member” increased significantly, indicating that processing time modulated the precision of individual representations. However, exposure time greater than 500 ms did not further increase the ratio of “yes” response to the members. Interestingly, the ratio of “yes” response to the “mean” faces also increased with more processing time available. Taken together, both individual and mean representation of emotional faces were enhanced with more processing time.

Results showed that participants inclined to make more “yes” response to Mean than Member. This pattern was stable across relatively brief exposure times (50 to 1500 ms). This supported the idea that participants unconsciously represent the mean information of a set of stimuli (Ariely, 2001; Haberman and Whitney, 2009). However, when exposure time was increased to 2000 ms, the ratios were no longer significantly different. This may suggest the mean representation becomes noisier as set duration increases to 2000 ms.

## Experiment 2

In member identification task, individual representations are emphasized, and mean representation is extracted implicitly. In Experiment 2, we adopted the mean discrimination paradigm to emphasize mean representation (Ariely, 2001), in which participants were explicitly asked to extract mean emotion from multiple faces. We manipulated the similarity among members of face set, where the faces were either homogeneous or heterogeneous. We hypothesized that participants would extract mean representation more accurately with longer exposure time, and there would be correlation between performance of individual and mean representations.

### Methods

#### Participants

Sixteen (age 20–26, seven males) undergraduate and graduate students participated in this experiment for a small payment. Informed consent was provided before the experiment. All are right-handed and have normal or corrected to normal vision.

#### Stimuli

Same as in Experiment 1 with an additional pool of homogenous stimuli set (used across conditions).

#### Design

Experiment 2 adopted a 2 × 4 within subject design. Set type (two levels: homogenous vs. heterogeneous) and exposure time (four levels: 50/500/1000/2000 ms) were manipulated as the two independent variables.

#### Procedure

**Figure 2B** illustrates the experimental procedure. After a fixation of 500 ms, a compare face was presented in the center of the display for 1500 ms, followed by a set of four faces, presented simultaneously. Participants were asked to judge whether the mean emotion of the four faces were more neutral than the previous compare face. The speed and accuracy of responses were both emphasized.

Following Haberman and Whitney (2009, Experiment 1B), four faces in the set were the same in the homogeneous set, but different from each other in the heterogeneous set. The setting of emotional intensities was the same as that in Experiment 1. In both homogeneous and heterogeneous conditions, the difference between compare face and the mean of set faces was ±10, ±8, ±4, or ±2 emotional units.

The eight conditions (two set types × four durations) were carried out in separate blocks with one condition per block. The order of the blocks was counterbalanced across participants. Each block consisted of 64 trials, and the trial order was randomized.

### Results

Two participants were excluded from analysis (one had reaction time exceeding two standard deviations of the grand mean, another misunderstood the task instruction), resulting in 14 participants data in the further analysis. Furthermore, data out of two standard deviations (2.4% of all data) were excluded from further analysis.

*A*′ (Pollack and Norman, 1964) was computed for each participant in each condition as an indicator of the discrimination power. *A*′ data was derived from the results of hit (H) and false alarm (F) rates:

$$\begin{array}{c} A^{\prime }=0.5+\left[\mathrm{sign}(H-F)\frac{{\left(H-F\right)}^{2}+|H-F|}{4\;\max(H,F)-4HF}\right] \end{array}$$

where sign(H - F) equals +1 if H > F, 0 if H = F, and -1 otherwise, and max(H, F) equals either H or F, whichever is greater. A′ ranges from 0.5 to 1, with 0.5 indicating no discrimination ability, and 1 indicating perfect discrimination ability. In the calculation of A′, the stimulus set being more neutral was denoted as the signal. Specifically, “yes” responses to the more neutral face sets were marked as hit, while “yes” responses to the less neutral face sets were marked as false alarm.

**FIGURE 4:**
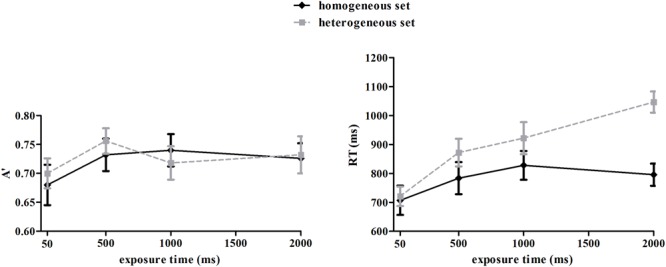
**Discriminating sensitivity A′ and reaction time (RT) as a function of duration and set type.** Error bars represent one standard error of the means.

As the Cox-Small test showed the A′ data violated the assumption of multivariate normality (*p* = 0.041), we applied arcsine square root transformation to the data. The Cox-Small test was used to assess the multivariate normality of the transformed A′ data, which showed a normal distribution (*p* = 0.243). Overall, participants were able to discriminate mean emotion of the face set from the compare face (**Figure 4**, left panel), as A′ was significantly higher than 0.5 (chance level), *t’*s(13) > 19.38, *p*s < 0.001. A repeated-measures ANOVA on transformed data revealed no significant effect of set type, *F*(1,13) = 0.131, *p* = 0.723, suggesting comparable discriminating ability for homogeneous and heterogeneous sets. The interaction between set type and exposure time was not significant, *F*(3,39) = 0.393, *p* = 0.759. However, there was a significant main effect of exposure time, *F*(3,39) = 3.02, *p* = 0.041, η_p_^2^ = 0.19. Multiple comparison showed that discrimination in the 50 ms condition was significantly lower than in the conditions of 500, 1000, and 2000 ms (*p*s < 0.05), and the latter three conditions were comparable (*p*s > 0.05). A correlation analysis showed that A′ of the homogeneous and heterogeneous sets were significantly correlated with each other in the longer duration conditions (500 ms: *r* = 0.66, *p* = 0.005; 1000 ms: *r* = 0.58, *p* = 0.015; 2000 ms: *r* = 0.45, *p* = 0.052), but not in the 50 ms condition (*r* = -0.23, *p* = 0.214). Furthermore, A′ in the 500, 1000, and 2000 ms conditions were significantly correlated with each other within both homogeneous (*r*s = 0.80, 0.76, 0.54, *p*s < 0.05) and heterogeneous (*r*s = 0.56, 0.54, 0.75, *p*s < 0.05) sets. These results suggested that the precision of individual and ensemble representations improved with longer exposure time, and was correlated with each other. However, A′ in the 500, 1000, and 2000 ms conditions were not correlated with A′ in the 50 ms condition (*r*s < 0.34, *p*s > 0.12). Taken together, these results might indicate that the mechanism of processing multiple faces in short duration was different from that in longer durations, for example, the ensemble representation in short duration may be more coarse, and not dependent on individual representations.

Reaction time data for the correct trials were plotted in **Figure 4** (right panel). The Cox-Small test of multivariate normality showed that the RT data were normally distributed (*p* = 0.209). There were significant main effects of set type, *F*(1,13) = 12.00, *p* < 0.01, η_p_^2^ = 0.48, and exposure time, *F*(3,39) = 13.01, *p* < 0.001, η_p_^2^ = 0.50. The interaction of the two factors was also significant *F*(3,39) = 11.32, *p* < 0.001, η_p_^2^ = 0.47. Simple effect analysis indicated that reaction times were influenced by exposure time for both the homogeneous set, *F*(3,39) = 4.22, *p* < 0.05, η_p_^2^ = 0.25 and the heterogeneous set, *F*(3,39) = 17.59, *p* < 0.001, η_p_^2^ = 0.58. However, the trends were different: for the homogeneous set, reaction times increased from 50 to 500 ms of exposure, and stabilized with longer exposure, while for the heterogeneous set, reaction times kept rising with the increased exposure time. When reaction times of the two sets in same exposure time were compared, we found no significant differences in the 50 and 500 ms conditions, but significant differences in the 1000 and 2000 ms conditions (*p*s < 0.05).

#### The Diffusion Model

Both reaction time analysis and the correlation analysis of A′ suggest that mechanisms for processing individual items and ensemble representation might be differently depending on whether the stimuli are presented for a short or a longer duration. This difference may be accounted for by more precise individual representations as a result of longer processing time. However, longer duration also introduces more noise to decision process, as noise is also accumulated in the accumulation process of decision information (Ratcliff and McKoon, 2008). It remains an open question how the noise affects the performance: does it slow down processing speed, or render response criterion more conservative? In order to investigate this issue, we adopted the diffusion model (Ratcliff, 1978; Ratcliff and McKoon, 2008), which decomposes the accuracy and reaction time data into distinct cognitive subcomponents.

The basic assumption of the diffusion model is that in a rapid two-alternative choice task, the information needed for making a choice accumulates from the starting point until it reaches the decision boundary of one of the choices. The model has four parameters describing the decision performance (see Ratcliff and McKoon, 2008, for more details):

(1) Drift rate, *v*, information accumulating rate, determined by the quality of the information extracted from the stimuli.
(2) Boundary separation, *a*, the amount of information needed to make a decision, sensitive to speed vs. accuracy instructions or decision criterion.
(2) Starting point, *z*, prior bias before decision making.
(4) Non-decisional time, *t*_0_, time for encoding, response execution, and other non-decisional process.

The hierarchical diffusion model (HDM, Vandekerckhove et al., 2011) was used to fit the data, because of its strength in considering individual differences. We assumed that there was no prior bias for the response and set the starting point at *a*/2. Other parameter were set to adjust with independent variables (set type, exposure time). The data was fed into the HDM analysis and acquired the drift rate (*v*), boundary separation (*a*), and non-decisional time (*t*_0_) for each participants in each condition.

**FIGURE 5:**
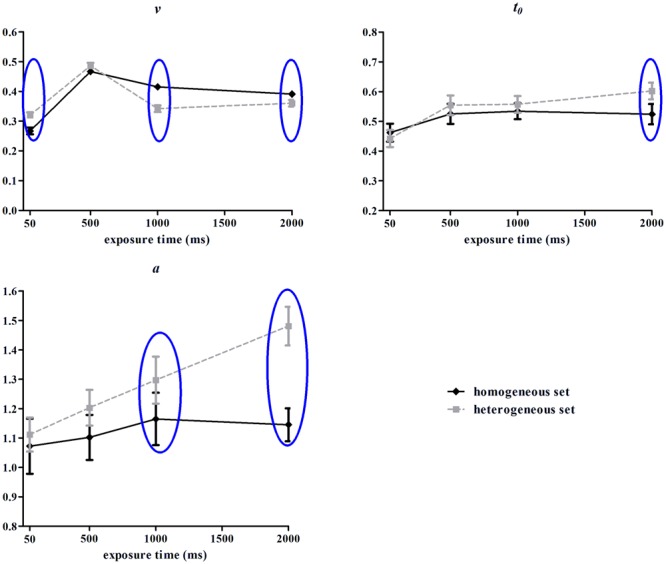
**Drift rate (*v*), boundary separation (*a*), and non-decisional time (*t*_0_) from hierarchical diffusion model (HDM) analysis in Experiment 2.** Error bars represent one standard error of the means. Blue circles indicate statistical significance (*p* < 0.05).

The Cox-Small test of multivariate normality showed that the parameters data (*a, v, t*_0_) were normally distributed (*p* = 0.877, 0.827, 0.124, respectively). These parameters were depictured in **Figure 5** and submitted to repeated-measures ANOVAs. The main effects of set type for *v* and *t*_0_ were insignificant, *F*s(1,13) = 1.16, 3.26, *p*s = 0.30,.09, η_p_^2^ = 0.08, 0.20, but was significant for *a, F*(1,13) = 9.00, *p* = 0.01, η_p_^2^ = 0.41. The main effects of exposure time for *v, a* and *t*_0_ were significant, *F*s(3,39) = 139.08, 5.52, 11.31, *p*s < 0.001, η_p_^2^ = 0.90, 0.30, 0.47. The interactions of set type and exposure time for *v, a* and *t*_0_ were significant, *F*s(3,39) = 15.82, 6.34, 3.78, *p*s < 0.05, η_p_^2^ = 0.55, 0.33, 0.23.

Simple effect analysis for drift rate (*v*) showed an inverse U curve as the function of exposure time, with the 500 ms condition as the turning point. This pattern found for both homogeneous set, *F*(3,39) = 99.44, *p* < 0.001, η_p_^2^ = 0.88, and heterogeneous set, *F*(3,39) = 52.56, *p* < 0.001, η_p_^2^ = 0.80. It also showed that drift rate for heterogeneous set was higher than for homogeneous set in 50 ms duration, *F*(1,13) = 9.93, *p* = 0.008, η_p_^2^ = 0.43; lower than in homogeneous set in 1000/2000 ms duration, *F*(1,13) = 33.33, 4.43, *p* < 0.001, =0.055, η_p_^2^ = 0.72, 0.25, but comparable in 500 ms duration, *F*(1,13) = 2.95, *p* = 0.11, η_p_^2^ = 0.18.

Simple effect analysis for boundary separation (*a*) showed that the homogeneous condition was not influenced by exposure time, *F*(3,39) < 1, *p* > 0.05, η_p_^2^ = 0.05, while the separation between boundaries in the heterogeneous condition increased monotonically as a function of exposure time, *F*(3,39) = 12.67, *p* < 0.001, η_p_^2^ = 0.49. In addition, *a* for heterogeneous set was higher than for homogeneous set in 1000 and 2000 ms duration (*p* < 0.05, *p* < 0.001).

Simple effect analysis for non-decisional time (*t*_0_) showed that when the stimulus duration was 50 ms, both homogeneous and heterogeneous conditions required the least non-decisional time and were significantly different from the conditions with longer exposure time, homogeneous: *F*(3,39) = 4.12, *p* < 0.05, η_p_^2^ = 0.24; heterogeneous: *F*(3,39) = 12.03, *p* < 0.001, η_p_^2^ = 0.48. However, it was only when the stimuli were presented for 2000 ms, did the heterogeneous condition required more non-decisional time than the homogeneous condition (*p* < 0.05).

### Discussion

Similar patterns of A′ in homogeneous and heterogeneous image conditions suggested that human observers could extract emotion information from multiple faces as precisely as from a single face. This confirms the previous findings from homogeneous image conditions (Haberman and Whitney, 2007, 2009). The speed of extracting emotional information from multiple faces was fast: mean information was extracted within 50 ms of stimulus exposure. This is consistent with results of previous research. For example, observers could accurately extract mean size of 12 dots of varying diameters within 50 ms of exposure time (Chong and Treisman, 2003).

Reaction time results showed distinct patterns in the homogeneous and the heterogeneous conditions. HDM analysis was applied to decompose the cognitive processes in the two conditions. According to Voss et al. (2013), drift rate (*v*) was related to task difficulty: the more difficult a task is, the less drift rate will be. One of our interesting findings was that the drift rate for the heterogeneous set was not lower than for the homogeneous set when they were shown in just 50 ms. The fact that observers accumulated information more quickly when the stimuli were heterogeneous is at first glance counterintuitive. However, Won and Jiang (2013) found a redundancy gain in the emotion processing of multiple faces in a brief presentation (200 ms). This raises a possibility of a redundancy gain for a heterogeneous set. Cohen et al. (2014) provided insight on this issue from a categorical overlap perspective. They showed that the ability to process multiple items at once (short processing time) is limited by the extent to which those items are represented by separate neural populations. Xu (2009) further provided that four heterogeneous objects activate stronger brain responses in the area of LOC (lateral occipital complex) and superior IPS (intraparietal sulcus) during object identification.

By comparing stimulus exposure time of 50 ms with 500 ms, we observed a clear rise of drift rate *v*, indicating that greater exposure time reduced the task difficulty. This may suggest a robust redundancy gain for processing multiple facial expressions. When stimulus exposure time was greater than 500 ms, the draft rate *v* began to decrease. Considering discrimination performance was not enhanced with time over 500 ms, this may suggest that a long exposure time could have introduced noise (Ratcliff and McKoon, 2008). This could have hindered task performance, although it could also induce a redundancy gain (Won and Jiang, 2013). The noise might lead to a conservative decision bias, as indicated in the boundary separation. Boundary separation (*a*) represents the amount of information needed for a decision, and it is related to observer’s decision style (Ratcliff et al., 2001). A conservative observer needs more information to make decisions, resulting in prolonged reaction time and enhanced accuracy. Boundary separation results suggested that exposure time did not affect the decision style for the homogeneous set; however, in the heterogeneous set, longer exposure time turned the participants into more conservative observers. It is possible that when time is limited, the sense of urgency lead the participants to lower the decision threshold and make decisions as soon as possible (Kira and Shadlen, 2010). Our results from diffusion model analysis confirmed this account, and showed that boundary separation was lower in 50 ms duration, and the drift rate for heterogeneous set was higher than for homogeneous set in 50 ms duration.

## General Discussion

The present study investigated the role of individual representations during processing multiple facial expressions with varying processing resource. Both Experiments 1 and 2 showed that human observers are capable of extracting and discriminating mean emotional information from multiple faces, and the precision of both individual and mean representations increased with more processing time available. Furthermore, our data suggested that the relationship between individual and mean representations also depended on the availability of processing time. Specifically, the precision of mean representation appears to be independent of individual representations at brief processing time, but more accurate individual representations are achieved at a longer processing time and improve mean representation. However, the precision of mean representation also suffers from the noise accumulated in redundant time.

### Time-Dependent Processing of Multiple Facial Expressions

Both Experiments 1 and 2 showed that when exposure time was limited to 50 ms, the response for mean representation was significantly superior to the response for set member. These results in both membership identification and mean emotion judgment tasks suggested robust mechanisms for coping with brief processing time to build an ensemble representation. Support for this idea can also be found in developmental prosopagnosia patients who lost the ability to process single faces but preserved the ability to represent mean emotion or identity from multiple faces (Leib et al., 2012).

When more processing time is allowed, the quality of both individual and ensemble representations is improved. However, there seems to be a turning point of set duration for individual expression processing (around 500 ms in our study) where individual processing become stable across longer durations. There is also a turning point of set duration for ensemble coding where ensemble representation begins to become coarser due to noise accumulated in the longer processing time (maybe redundant). These different trends of individual and mean representations varying with exposure times indicated mean representation was not a simple linear relationship with individual representations. This suggested that it was not a rivalry relationship between individual and mean representations, and may be established by two separate mechanisms. One possible explanation is that the representation for multiple faces has a hierarchical structure that representations of different levels were stored at the same time (Brady and Alvarez, 2011).

We suggest that the mechanism of processing multiple facial expressions may depend on the availability of processing time, which could be defined into three types of time availability: scarce, sufficient, and redundant. When time is scarce, there can be no precise individual representations, so the only solution is to rely on the more global, mean representation. However, when time is sufficient, individual representations are built and refined to gain more knowledge of the stimuli set. The precision of both individual and mean representations improves while relatively more weights are given to individual representations compared to a scarce condition. When time is redundant, however, irrelevant noises could accumulate to damage the quality of processing and induce a conservative decision bias (Ratcliff and McKoon, 2008). Although it is unknown about how the relative weights change in the final representation, the present study confirms that the availability of processing resources modulate the relationship of individual and averaging during ensemble coding.

### The Relationship of Individual and Ensemble Representations

Individual and ensemble representations are not used in isolation. Mean representation of faces can compensate for the imprecise nature of individual representations when processing resource is limited. Previous studies suggested that extraction of ensemble representation may be an automatic process without computing precise individual representations (Chong and Treisman, 2003; Haberman and Whitney, 2009, 2011). Alvarez (2011) pointed out that when multiple items are averaged together, the random noise in individual representations could counteract each other to achieve a more accurate ensemble representation. This was validated by Haberman and Whitney (2011) who showed that when the expression of a face changes in a crowd of faces, participants were not aware of this change, but could still extract accurate mean emotion from the face crowd. These studies often presented a stimulus set in a brief duration that can be beyond the processing capacity of human observers if the individual items had to be processed in a serial fashion. Based on the fairly good performance on the estimation of the mean in a stimulus set, these studies were able to support the element-independent assumption of ensemble representation. Our data for 50 ms duration was consistent with this claim.

However, our data also showed that ensemble representation depend on the precision of individual representations, which could be improved with more available processing time. Haberman and Whitney (2009, Experiment 2) also showed that the mean emotion representation becomes more precise as set duration increases, indicated by the narrower width of the Gaussian fit. These results seemed to indicate that ensemble coding is an adaptive process, which relies on available processing resources. In this process, the global ensemble representation has a priority over representations of individual items. That is, when the visual system attempts to accomplish ensemble coding under time pressure, it recourses to a coarse sketch of ensemble at the expense of individual representations. However, when the visual system is given sufficient time it will start to build up more detailed individual representations, which also result in a more precise ensemble representation. Like in scene perception, where the gist is automatically encoded separately from specific features (Sampanes et al., 2008), an ensemble representation may also be created automatically. Thus there may be two separate mechanisms for constructing individual and ensemble representations. Our results provide evidence for the two mechanisms by demonstrating resource-dependent processing of a complex set of emotional faces. This adds to the existing evidence that a precise individual representation can be constructed and contribute to a mean representation if the individual items required less processing resources (Neumann et al., 2013).

### Individual and Mean Representation Serve Different Social Functions

Membership identification and mean discrimination tasks may serve two distinct social functions that have an individual-orientation or crowd-orientation. For instance, picking a friend up at a train station involves searching for a particular face. This relies on an individual representation, but not the ensemble coding. In contrast, the enjoyment of watching a group performance could be impaired if one focuses only on a few performers and lose the whole view.

We encounter different types of social scenarios, so our strategies can be fluid and flexible. Results of this study showed that in an individual-oriented task like membership identification, individual representation played an important role in the completing the task; while in a crowd-oriented task like judging the mean emotion of a face set, the process for creating an ensemble representation takes precedence over building individual representations.

## Author Contributions

LJ, HL, and WC contributed to the design of the work. LJ and HL contributed to the acquisition of data. LJ and KT contributed to the analysis, or interpretation of the data and drafted the paper. WC and CL revised it critically. All the authors reviewed and commented on the draft and approved this final version to be published.

## Conflict of Interest Statement

The authors declare that the research was conducted in the absence of any commercial or financial relationships that could be construed as a potential conflict of interest.
